# Diethyl 3-amino-4-[(triphenyl­phosphor­anyl­idene)amino]thieno[2,3-*b*]thio­phene-2,5-dicarboxyl­ate

**DOI:** 10.1107/S1600536808015705

**Published:** 2008-06-07

**Authors:** Xiang Wang, Yan Li, Ming-Guo Liu

**Affiliations:** a Department of Chemistry, Kaili College, Kaili 556000, People’s Republic of China; b Department of Chemistry and Life Science, Xianning College, Xianning 437000, People’s Republic of China; c Hubei Key Laboratory of Natural Products Research and Development, China Three Gorges University, Yichang 443002, People’s Republic of China

## Abstract

The asymmetric unit of the title compound, C_30_H_27_N_2_O_4_PS_2_, consists of two crystallographically independent mol­ecules. The thieno[2,3-*b*]thio­phene ring systems are planar. One of the terminal ethyl groups is disordered over two positions; the site occupancy factors are *ca* 0.7 and 0.3. The crystal structure is stabilized by N—H⋯N, N—H⋯O and C—H⋯O hydrogen bonds.

## Related literature

Related preparation and biological activity are described by Walter (1999*a* ▶,*b* ▶). For related literature, see: Ding *et al.* (2004 ▶). For the crystal structure of another fused pyrimidinone derivative, see: Liu & Hu (2006 ▶). For related literature, see: Liao *et al.* (2007 ▶).
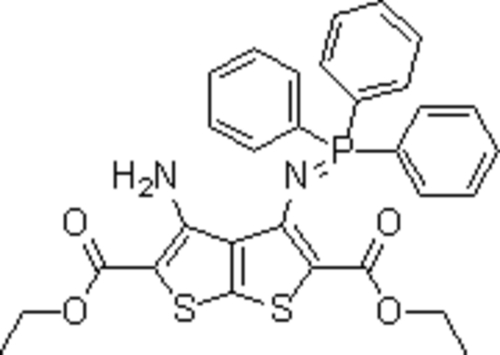


## Experimental

### 

#### Crystal data

C_30_H_27_N_2_O_4_PS_2_
*M* *_r_* = 574.63
Triclinic, 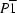

*a* = 13.1260 (9) Å
*b* = 15.3616 (10) Å
*c* = 16.6497 (11) Å
α = 68.140 (1)°
β = 71.043 (1)°
γ = 68.969 (1)°
*V* = 2836.5 (3) Å^3^
*Z* = 4
Mo *K*α radiation
μ = 0.28 mm^−1^
*T* = 298 (2) K
0.20 × 0.10 × 0.06 mm

#### Data collection

Bruker SMART 4K CCD area-detector diffractometer
Absorption correction: multi-scan (*SADABS*; Bruker, 2001 ▶) *T*_min_ = 0.946, *T*_max_ = 0.983
20968 measured reflections
10981 independent reflections
6314 reflections with *I* > 2σ(*I*)
*R*_int_ = 0.053

#### Refinement

*R*[*F* ^2^ > 2σ(*F* ^2^)] = 0.068
*wR*(*F* ^2^) = 0.159
*S* = 0.96
10981 reflections
727 parameters
6 restraints
H-atom parameters constrained
Δρ_max_ = 0.48 e Å^−3^
Δρ_min_ = −0.28 e Å^−3^

### 

Data collection: *SMART* (Bruker, 2001 ▶); cell refinement: *SAINT-Plus* (Bruker, 2001 ▶); data reduction: *SAINT-Plus*; program(s) used to solve structure: *SHELXS97* (Sheldrick, 2008 ▶); program(s) used to refine structure: *SHELXL97* (Sheldrick, 2008 ▶); molecular graphics: *PLATON* (Spek, 2003 ▶); software used to prepare material for publication: *SHELXTL* (Sheldrick, 2008 ▶).

## Supplementary Material

Crystal structure: contains datablocks I, global. DOI: 10.1107/S1600536808015705/bt2711sup1.cif

Structure factors: contains datablocks I. DOI: 10.1107/S1600536808015705/bt2711Isup2.hkl

Additional supplementary materials:  crystallographic information; 3D view; checkCIF report

## Figures and Tables

**Table 1:** Hydrogen-bond geometry (Å, °)

*D*—H⋯*A*	*D*—H	H⋯*A*	*D*⋯*A*	*D*—H⋯*A*
C33—H33⋯O3	0.93	2.46	3.298 (5)	151
N4—H4*B*⋯O7	0.86	2.16	2.817 (4)	134
N4—H4*A*⋯N3	0.86	2.29	2.945 (4)	133
N2—H2*B*⋯O3	0.86	2.19	2.832 (4)	131
N2—H2*A*⋯N1	0.86	2.33	2.965 (4)	131

**Fig. 1.**
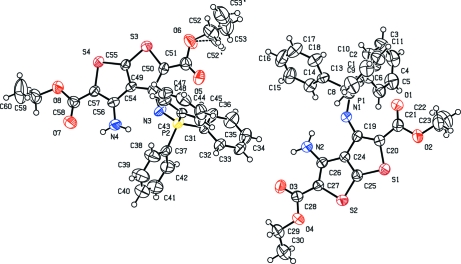
Molecular structure of the title compound with labelling and displacement ellipsoids drawn at the 50% probability level.

## supplementary crystallographic information

### Comment

The derivatives of heterocycles containing the thienopyrimidine system, which are well known bioisosteres of quinazolines, are of great importance because of their remarkable biological properties (Walter, 1999a,b; Ding *et al.*, 2004). As a part of our ongoing investigations on the preparation of derivatives of heterocyclic compounds (Liu & Hu, 2006; Liao *et al.*, 2007), we have synthesized and structurally characterized characterized the title compound. In the crystal structure of the title compound two crystallographically independent molecules are found in the asymmetric unit. All ring atoms of the thieno[2,3-*b*]thiophene ring systems lie in a common plane, with maximum deviations of 0.01 (1) Å and 0.02 (1) Å. The crystal structure is stabilized by N—H···N, N—H···O and C—H···O hydrogen bonds (Table 1).

### Refinement

One of the terminal ethyl groups is disordered over two sites with site occupation factors of 0.66 (2) and 0.34 (2). The following restraints have been applied for the disordered atoms: O—CH_2_ 1.45 (1) Å, CH_2_—CH_3_ 1.54 (1) Å and O···CH_3_ 2.45 (1) Å. All H-atoms were positioned with idealized geometry and refined with fixed isotropic U values (*U*_iso_(H) = 1.5*U*_eq_(C) for methyl H atoms and *U*_iso_(H) = 1.2*U*_eq_(C) for all other H atoms) using a riding model with C—H = 0.93 Å, 0.97 Å = 0.96 Å and N—H = 0.86 Å.

### Crystal data

**Table:**

C_30_H_27_N_2_O_4_PS_2_	*Z* = 4
*M**_r_* = 574.63	*F*_000_ = 1200
Triclinic, *P*1	*D*_x_ = 1.346 Mg m^−^^3^
Hall symbol: -P 1	Mo *K*α radiation λ = 0.71073 Å
*a* = 13.1260 (9) Å	Cell parameters from 2577 reflections
*b* = 15.3616 (10) Å	θ = 2.3–19.7º
*c* = 16.6497 (11) Å	µ = 0.28 mm^−^^1^
α = 68.1400 (10)º	*T* = 298 (2) K
β = 71.0430 (10)º	Block, yellow
γ = 68.9690 (10)º	0.20 × 0.10 × 0.06 mm
*V* = 2836.5 (3) Å^3^	

### Data collection

**Table:**

Bruker SMART 4K CCD area-detector diffractometer	10981 independent reflections
Radiation source: fine-focus sealed tube	6314 reflections with *I* > 2σ(*I*)
Monochromator: graphite	*R*_int_ = 0.053
*T* = 298(2) K	θ_max_ = 26.0º
φ and ω scans	θ_min_ = 1.5º
Absorption correction: multi-scan(SADABS; Bruker, 2001)	*h* = −15→16
*T*_min_ = 0.946, *T*_max_ = 0.983	*k* = −18→18
20968 measured reflections	*l* = −14→20

### Refinement

**Table:**

Refinement on *F*^2^	Secondary atom site location: difference Fourier map
Least-squares matrix: full	Hydrogen site location: inferred from neighbouring sites
*R*[*F*^2^ > 2σ(*F*^2^)] = 0.068	H-atom parameters constrained
*wR*(*F*^2^) = 0.159	*w* = 1/[σ^2^(*F*_o_^2^) + (0.0624*P*)^2^] where *P* = (*F*_o_^2^ + 2*F*_c_^2^)/3
*S* = 0.96	(Δ/σ)_max_ = 0.001
10981 reflections	Δρ_max_ = 0.48 e Å^−^^3^
727 parameters	Δρ_min_ = −0.28 e Å^−^^3^
6 restraints	Extinction correction: none
Primary atom site location: structure-invariant direct methods	

### Special details

**Table:**

Geometry. All e.s.d.'s (except the e.s.d. in the dihedral angle between two l.s. planes) are estimated using the full covariance matrix. The cell e.s.d.'s are taken into account individually in the estimation of e.s.d.'s in distances, angles and torsion angles; correlations between e.s.d.'s in cell parameters are only used when they are defined by crystal symmetry. An approximate (isotropic) treatment of cell e.s.d.'s is used for estimating e.s.d.'s involving l.s. planes.
Refinement. Refinement of *F*^2^ against ALL reflections. The weighted *R*-factor *wR* and goodness of fit *S* are based on *F*^2^, conventional *R*-factors *R* are based on *F*, with *F* set to zero for negative *F*^2^. The threshold expression of *F*^2^ > σ(*F*^2^) is used only for calculating *R*-factors(gt) *etc*. and is not relevant to the choice of reflections for refinement. *R*-factors based on *F*^2^ are statistically about twice as large as those based on *F*, and *R*- factors based on ALL data will be even larger.

### Fractional atomic coordinates and isotropic or equivalent isotropic displacement parameters (Å^2^)

**Table:**

	*x*	*y*	*z*	*U*_iso_*/*U*_eq_	Occ. (<1)
C1	0.2610 (3)	0.4257 (3)	0.8472 (2)	0.0461 (9)	
C2	0.2866 (3)	0.4036 (3)	0.9289 (3)	0.0608 (11)	
H2	0.2896	0.4528	0.9471	0.073*	
C3	0.3074 (4)	0.3091 (3)	0.9823 (3)	0.0705 (13)	
H3	0.3214	0.2951	1.0378	0.085*	
C4	0.3078 (4)	0.2357 (3)	0.9548 (4)	0.0772 (15)	
H4	0.3238	0.1717	0.9909	0.093*	
C5	0.2847 (4)	0.2559 (3)	0.8746 (3)	0.0759 (14)	
H5	0.2846	0.2057	0.8562	0.091*	
C6	0.2617 (3)	0.3504 (3)	0.8208 (3)	0.0609 (12)	
H6	0.2465	0.3636	0.7660	0.073*	
C7	0.1246 (3)	0.6316 (3)	0.8246 (2)	0.0464 (9)	
C8	0.0934 (3)	0.7258 (3)	0.7712 (3)	0.0639 (12)	
H8	0.1283	0.7422	0.7114	0.077*	
C9	0.0103 (4)	0.7958 (3)	0.8068 (3)	0.0803 (15)	
H9	−0.0104	0.8592	0.7705	0.096*	
C10	−0.0415 (4)	0.7735 (3)	0.8939 (4)	0.0783 (14)	
H10	−0.0966	0.8214	0.9173	0.094*	
C11	−0.0122 (4)	0.6801 (4)	0.9473 (3)	0.0740 (14)	
H11	−0.0475	0.6644	1.0071	0.089*	
C12	0.0699 (3)	0.6089 (3)	0.9126 (3)	0.0591 (11)	
H12	0.0883	0.5452	0.9488	0.071*	
C13	0.3626 (3)	0.5803 (3)	0.7625 (3)	0.0474 (10)	
C14	0.4623 (3)	0.5437 (3)	0.7082 (3)	0.0580 (11)	
H14	0.4629	0.5054	0.6759	0.070*	
C15	0.5600 (4)	0.5627 (3)	0.7009 (4)	0.0758 (14)	
H15	0.6260	0.5372	0.6644	0.091*	
C16	0.5595 (5)	0.6190 (4)	0.7476 (4)	0.0951 (18)	
H16	0.6252	0.6328	0.7423	0.114*	
C17	0.4628 (5)	0.6557 (4)	0.8025 (4)	0.0954 (17)	
H17	0.4638	0.6931	0.8350	0.114*	
C18	0.3640 (4)	0.6374 (3)	0.8099 (3)	0.0712 (13)	
H18	0.2985	0.6633	0.8465	0.085*	
C19	0.1617 (3)	0.5670 (2)	0.6309 (2)	0.0390 (9)	
C20	0.0575 (3)	0.5474 (3)	0.6605 (2)	0.0466 (9)	
C21	0.0036 (3)	0.5049 (3)	0.7492 (3)	0.0523 (10)	
C22	−0.1442 (4)	0.4291 (4)	0.8356 (3)	0.106 (2)	
H22A	−0.2206	0.4370	0.8349	0.127*	
H22B	−0.1461	0.4526	0.8829	0.127*	
C23	−0.0812 (6)	0.3255 (5)	0.8516 (4)	0.156 (3)	
H23A	−0.0794	0.3029	0.8044	0.233*	
H23B	−0.1171	0.2882	0.9070	0.233*	
H23C	−0.0062	0.3181	0.8535	0.233*	
C24	0.1894 (3)	0.5986 (2)	0.5359 (2)	0.0375 (8)	
C25	0.1080 (3)	0.6043 (2)	0.4975 (2)	0.0460 (9)	
C26	0.2835 (3)	0.6265 (2)	0.4709 (2)	0.0402 (9)	
C27	0.2694 (3)	0.6522 (2)	0.3857 (2)	0.0429 (9)	
N2	0.3742 (3)	0.6258 (2)	0.4942 (2)	0.0568 (9)	
H2A	0.3759	0.6025	0.5494	0.068*	
H2B	0.4255	0.6458	0.4504	0.068*	
C28	0.3516 (3)	0.6807 (3)	0.3064 (3)	0.0508 (10)	
C29	0.3981 (4)	0.7238 (4)	0.1496 (3)	0.0856 (16)	
H29A	0.3780	0.7941	0.1241	0.103*	
H29B	0.4725	0.7034	0.1607	0.103*	
C30	0.3979 (5)	0.6781 (4)	0.0880 (3)	0.111 (2)	
H30A	0.4189	0.6086	0.1129	0.166*	
H30B	0.4503	0.6963	0.0333	0.166*	
H30C	0.3243	0.6989	0.0767	0.166*	
C31	0.7449 (3)	0.8334 (3)	0.3607 (3)	0.0460 (9)	
C32	0.6524 (3)	0.8387 (3)	0.3338 (3)	0.0507 (10)	
H32	0.6151	0.8976	0.2995	0.061*	
C33	0.6157 (3)	0.7574 (3)	0.3577 (3)	0.0583 (11)	
H33	0.5528	0.7620	0.3404	0.070*	
C34	0.6709 (4)	0.6700 (3)	0.4066 (3)	0.0672 (13)	
H34	0.6462	0.6151	0.4216	0.081*	
C35	0.7622 (4)	0.6624 (3)	0.4337 (3)	0.0756 (14)	
H35	0.7987	0.6027	0.4677	0.091*	
C36	0.8008 (3)	0.7439 (3)	0.4105 (3)	0.0639 (12)	
H36	0.8635	0.7385	0.4283	0.077*	
C37	0.8032 (3)	0.9893 (3)	0.2095 (3)	0.0467 (9)	
C38	0.7843 (4)	1.0884 (3)	0.1696 (3)	0.0755 (14)	
H38	0.7583	1.1301	0.2048	0.091*	
C39	0.8030 (5)	1.1264 (4)	0.0797 (3)	0.0946 (17)	
H39	0.7896	1.1935	0.0546	0.114*	
C40	0.8409 (4)	1.0675 (5)	0.0260 (3)	0.0891 (16)	
H40	0.8532	1.0939	−0.0353	0.107*	
C41	0.8603 (4)	0.9702 (5)	0.0631 (4)	0.0893 (16)	
H41	0.8871	0.9295	0.0268	0.107*	
C42	0.8409 (4)	0.9297 (4)	0.1547 (3)	0.0713 (13)	
H42	0.8532	0.8626	0.1792	0.086*	
C43	0.6981 (3)	1.0324 (2)	0.3743 (2)	0.0402 (9)	
C44	0.5824 (3)	1.0483 (3)	0.3927 (3)	0.0601 (11)	
H44	0.5530	1.0056	0.3855	0.072*	
C45	0.5117 (4)	1.1272 (3)	0.4216 (3)	0.0675 (13)	
H45	0.4346	1.1377	0.4337	0.081*	
C46	0.5541 (4)	1.1899 (3)	0.4326 (3)	0.0674 (13)	
H46	0.5058	1.2433	0.4517	0.081*	
C47	0.6676 (4)	1.1749 (3)	0.4157 (3)	0.0595 (11)	
H47	0.6962	1.2175	0.4240	0.071*	
C48	0.7390 (3)	1.0968 (3)	0.3864 (2)	0.0509 (10)	
H48	0.8160	1.0871	0.3746	0.061*	
C49	0.9839 (3)	0.9001 (2)	0.3903 (2)	0.0395 (9)	
C50	0.9597 (3)	0.8761 (2)	0.4826 (3)	0.0422 (9)	
C51	0.8587 (3)	0.8594 (3)	0.5433 (3)	0.0538 (11)	
C52	0.7663 (10)	0.8289 (6)	0.7033 (9)	0.081 (4)	0.66 (2)
H52A	0.6978	0.8565	0.6817	0.097*	0.66 (2)
H52B	0.7626	0.8626	0.7436	0.097*	0.66 (2)
C53	0.7848 (11)	0.7206 (7)	0.7484 (9)	0.106 (5)	0.66 (2)
H53A	0.7859	0.6891	0.7078	0.159*	0.66 (2)
H53B	0.7253	0.7096	0.7996	0.159*	0.66 (2)
H53C	0.8550	0.6943	0.7662	0.159*	0.66 (2)
C52'	0.774 (2)	0.788 (3)	0.6825 (9)	0.081 (8)	0.34 (2)
H52C	0.7843	0.7311	0.6660	0.097*	0.34 (2)
H52D	0.7023	0.8332	0.6734	0.097*	0.34 (2)
C53'	0.781 (2)	0.760 (2)	0.7789 (8)	0.112 (9)	0.34 (2)
H53D	0.8497	0.7107	0.7882	0.167*	0.34 (2)
H53E	0.7188	0.7353	0.8170	0.167*	0.34 (2)
H53F	0.7791	0.8162	0.7925	0.167*	0.34 (2)
C54	1.0954 (3)	0.9101 (2)	0.3543 (2)	0.0375 (8)	
C55	1.1516 (3)	0.8942 (2)	0.4175 (2)	0.0400 (9)	
C56	1.1601 (3)	0.9351 (2)	0.2662 (2)	0.0408 (9)	
C57	1.2648 (3)	0.9386 (2)	0.2658 (2)	0.0446 (9)	
N4	1.1221 (3)	0.9535 (2)	0.1935 (2)	0.0571 (9)	
H4A	1.0559	0.9473	0.2040	0.068*	
H4B	1.1662	0.9747	0.1446	0.068*	
C58	1.3466 (3)	0.9665 (3)	0.1870 (3)	0.0515 (10)	
C59	1.5255 (3)	1.0003 (4)	0.1294 (3)	0.0823 (16)	
H59A	1.5148	0.9979	0.0753	0.099*	
H59B	1.5997	0.9596	0.1371	0.099*	
C60	1.5155 (5)	1.1006 (4)	0.1224 (5)	0.142 (3)	
H60A	1.4414	1.1403	0.1160	0.214*	
H60B	1.5693	1.1252	0.0716	0.214*	
H60C	1.5292	1.1022	0.1750	0.214*	
N1	0.2389 (2)	0.5576 (2)	0.67443 (19)	0.0448 (8)	
O1	0.0358 (2)	0.4854 (2)	0.81614 (18)	0.0624 (8)	
O2	−0.0900 (2)	0.4850 (2)	0.75083 (18)	0.0763 (9)	
O3	0.4411 (2)	0.6868 (2)	0.30673 (18)	0.0635 (8)	
O4	0.3190 (2)	0.6972 (2)	0.23237 (18)	0.0648 (8)	
N3	0.9225 (2)	0.9157 (2)	0.3315 (2)	0.0482 (8)	
O5	0.7744 (2)	0.8654 (2)	0.52373 (19)	0.0656 (8)	
O6	0.8661 (2)	0.8356 (2)	0.6283 (2)	0.0808 (10)	
O7	1.3321 (2)	0.9920 (2)	0.1115 (2)	0.0711 (9)	
O8	1.4417 (2)	0.9638 (2)	0.20472 (18)	0.0662 (8)	
P1	0.23784 (8)	0.54841 (7)	0.77260 (7)	0.0440 (3)	
P2	0.79766 (8)	0.93793 (7)	0.32758 (7)	0.0426 (3)	
S1	−0.00477 (8)	0.56927 (8)	0.57338 (7)	0.0555 (3)	
S2	0.14118 (8)	0.64202 (8)	0.38364 (7)	0.0533 (3)	
S3	1.07321 (8)	0.86553 (7)	0.52315 (6)	0.0502 (3)	
S4	1.28460 (8)	0.90910 (8)	0.37331 (7)	0.0526 (3)	

### Atomic displacement parameters (Å^2^)

**Table:**

	*U*^11^	*U*^22^	*U*^33^	*U*^12^	*U*^13^	*U*^23^
C1	0.048 (2)	0.048 (2)	0.040 (2)	−0.0129 (18)	−0.0072 (18)	−0.0120 (18)
C2	0.067 (3)	0.059 (3)	0.054 (3)	−0.012 (2)	−0.018 (2)	−0.015 (2)
C3	0.075 (3)	0.068 (3)	0.050 (3)	−0.011 (3)	−0.016 (2)	−0.003 (2)
C4	0.064 (3)	0.055 (3)	0.084 (4)	−0.007 (2)	−0.016 (3)	0.003 (3)
C5	0.089 (4)	0.045 (3)	0.091 (4)	−0.019 (2)	−0.017 (3)	−0.017 (3)
C6	0.068 (3)	0.049 (3)	0.063 (3)	−0.017 (2)	−0.009 (2)	−0.017 (2)
C7	0.050 (2)	0.046 (2)	0.046 (2)	−0.0141 (18)	−0.0124 (19)	−0.0133 (18)
C8	0.070 (3)	0.053 (3)	0.053 (3)	−0.008 (2)	−0.007 (2)	−0.012 (2)
C9	0.078 (3)	0.050 (3)	0.087 (4)	−0.003 (2)	−0.005 (3)	−0.017 (3)
C10	0.076 (3)	0.063 (3)	0.094 (4)	−0.014 (3)	0.001 (3)	−0.042 (3)
C11	0.068 (3)	0.087 (4)	0.063 (3)	−0.020 (3)	0.005 (2)	−0.036 (3)
C12	0.063 (3)	0.057 (3)	0.051 (3)	−0.009 (2)	−0.008 (2)	−0.019 (2)
C13	0.056 (2)	0.044 (2)	0.049 (2)	−0.0127 (19)	−0.023 (2)	−0.0117 (19)
C14	0.055 (3)	0.049 (2)	0.071 (3)	−0.011 (2)	−0.020 (2)	−0.017 (2)
C15	0.054 (3)	0.070 (3)	0.101 (4)	−0.020 (2)	−0.023 (3)	−0.014 (3)
C16	0.076 (4)	0.102 (4)	0.125 (5)	−0.045 (3)	−0.037 (4)	−0.019 (4)
C17	0.106 (5)	0.111 (4)	0.107 (5)	−0.052 (4)	−0.036 (4)	−0.041 (4)
C18	0.076 (3)	0.075 (3)	0.081 (3)	−0.022 (3)	−0.029 (3)	−0.032 (3)
C19	0.042 (2)	0.0315 (18)	0.048 (2)	−0.0106 (16)	−0.0103 (18)	−0.0157 (16)
C20	0.048 (2)	0.050 (2)	0.043 (2)	−0.0181 (18)	−0.0085 (18)	−0.0119 (18)
C21	0.046 (2)	0.054 (2)	0.056 (3)	−0.018 (2)	−0.008 (2)	−0.013 (2)
C22	0.088 (4)	0.137 (5)	0.084 (4)	−0.074 (4)	−0.026 (3)	0.024 (4)
C23	0.197 (8)	0.157 (7)	0.116 (6)	−0.106 (6)	−0.051 (5)	0.027 (5)
C24	0.039 (2)	0.0314 (18)	0.041 (2)	−0.0080 (16)	−0.0125 (17)	−0.0077 (16)
C25	0.041 (2)	0.045 (2)	0.048 (2)	−0.0110 (17)	−0.0105 (18)	−0.0098 (18)
C26	0.044 (2)	0.0341 (19)	0.047 (2)	−0.0090 (16)	−0.0127 (18)	−0.0153 (17)
C27	0.047 (2)	0.044 (2)	0.042 (2)	−0.0147 (17)	−0.0108 (18)	−0.0136 (17)
N2	0.056 (2)	0.079 (2)	0.044 (2)	−0.0329 (18)	−0.0101 (16)	−0.0139 (17)
C28	0.058 (3)	0.055 (2)	0.046 (3)	−0.021 (2)	−0.008 (2)	−0.019 (2)
C29	0.101 (4)	0.119 (4)	0.051 (3)	−0.069 (3)	0.007 (3)	−0.022 (3)
C30	0.121 (5)	0.157 (6)	0.065 (4)	−0.082 (4)	0.013 (3)	−0.031 (4)
C31	0.039 (2)	0.047 (2)	0.059 (3)	−0.0080 (17)	−0.0128 (19)	−0.0254 (19)
C32	0.047 (2)	0.057 (2)	0.059 (3)	−0.0169 (19)	−0.015 (2)	−0.022 (2)
C33	0.051 (2)	0.072 (3)	0.071 (3)	−0.024 (2)	−0.011 (2)	−0.036 (2)
C34	0.065 (3)	0.054 (3)	0.094 (4)	−0.023 (2)	−0.005 (3)	−0.038 (3)
C35	0.077 (3)	0.043 (3)	0.109 (4)	−0.005 (2)	−0.033 (3)	−0.025 (3)
C36	0.056 (3)	0.048 (2)	0.099 (4)	−0.006 (2)	−0.032 (2)	−0.028 (2)
C37	0.033 (2)	0.064 (3)	0.052 (3)	−0.0135 (18)	−0.0104 (18)	−0.025 (2)
C38	0.104 (4)	0.073 (3)	0.048 (3)	−0.022 (3)	−0.014 (3)	−0.020 (2)
C39	0.137 (5)	0.089 (4)	0.057 (4)	−0.035 (4)	−0.023 (3)	−0.015 (3)
C40	0.100 (4)	0.116 (5)	0.057 (3)	−0.047 (4)	−0.012 (3)	−0.019 (3)
C41	0.099 (4)	0.121 (5)	0.065 (4)	−0.042 (4)	0.006 (3)	−0.054 (3)
C42	0.074 (3)	0.084 (3)	0.066 (3)	−0.026 (3)	−0.003 (3)	−0.039 (3)
C43	0.039 (2)	0.047 (2)	0.036 (2)	−0.0132 (17)	−0.0080 (16)	−0.0136 (17)
C44	0.049 (3)	0.061 (3)	0.069 (3)	−0.020 (2)	−0.006 (2)	−0.019 (2)
C45	0.045 (3)	0.068 (3)	0.074 (3)	−0.011 (2)	−0.002 (2)	−0.018 (3)
C46	0.078 (3)	0.055 (3)	0.055 (3)	−0.006 (2)	−0.005 (2)	−0.021 (2)
C47	0.074 (3)	0.054 (3)	0.062 (3)	−0.014 (2)	−0.019 (2)	−0.029 (2)
C48	0.053 (2)	0.049 (2)	0.057 (3)	−0.0088 (19)	−0.017 (2)	−0.024 (2)
C49	0.039 (2)	0.0315 (18)	0.052 (3)	−0.0061 (16)	−0.0138 (18)	−0.0168 (17)
C50	0.037 (2)	0.039 (2)	0.052 (3)	−0.0110 (16)	−0.0116 (18)	−0.0114 (17)
C51	0.047 (3)	0.051 (2)	0.058 (3)	−0.013 (2)	−0.012 (2)	−0.010 (2)
C52	0.079 (6)	0.084 (7)	0.064 (8)	−0.014 (5)	−0.003 (6)	−0.024 (5)
C53	0.116 (8)	0.099 (8)	0.114 (12)	−0.054 (7)	0.004 (8)	−0.044 (7)
C52'	0.076 (12)	0.12 (2)	0.038 (10)	−0.050 (16)	0.017 (9)	−0.013 (12)
C53'	0.139 (18)	0.11 (2)	0.087 (15)	−0.060 (18)	−0.008 (14)	−0.013 (14)
C54	0.039 (2)	0.0356 (19)	0.045 (2)	−0.0086 (16)	−0.0151 (18)	−0.0156 (16)
C55	0.0346 (19)	0.041 (2)	0.047 (2)	−0.0129 (16)	−0.0118 (17)	−0.0102 (17)
C56	0.041 (2)	0.042 (2)	0.044 (2)	−0.0057 (17)	−0.0170 (18)	−0.0161 (17)
C57	0.042 (2)	0.048 (2)	0.048 (2)	−0.0153 (18)	−0.0134 (18)	−0.0128 (18)
N4	0.0465 (19)	0.082 (2)	0.047 (2)	−0.0200 (17)	−0.0123 (16)	−0.0198 (18)
C58	0.051 (2)	0.052 (2)	0.048 (3)	−0.0124 (19)	−0.013 (2)	−0.011 (2)
C59	0.046 (3)	0.122 (4)	0.062 (3)	−0.034 (3)	−0.003 (2)	−0.005 (3)
C60	0.099 (5)	0.097 (5)	0.192 (7)	−0.051 (4)	−0.037 (5)	0.030 (4)
N1	0.0485 (18)	0.0506 (18)	0.0420 (19)	−0.0201 (15)	−0.0104 (15)	−0.0138 (15)
O1	0.0661 (19)	0.080 (2)	0.0462 (18)	−0.0334 (15)	−0.0117 (15)	−0.0117 (15)
O2	0.0663 (19)	0.108 (2)	0.0554 (19)	−0.0536 (18)	−0.0124 (15)	0.0029 (17)
O3	0.0550 (18)	0.083 (2)	0.065 (2)	−0.0353 (16)	−0.0010 (15)	−0.0294 (16)
O4	0.0712 (19)	0.090 (2)	0.0372 (17)	−0.0402 (16)	−0.0035 (14)	−0.0119 (15)
N3	0.0374 (17)	0.063 (2)	0.058 (2)	−0.0117 (15)	−0.0158 (15)	−0.0293 (17)
O5	0.0432 (17)	0.077 (2)	0.078 (2)	−0.0220 (15)	−0.0091 (15)	−0.0223 (16)
O6	0.0546 (18)	0.111 (3)	0.053 (2)	−0.0268 (17)	−0.0046 (15)	−0.0002 (18)
O7	0.0635 (19)	0.094 (2)	0.0507 (19)	−0.0274 (16)	−0.0103 (15)	−0.0122 (17)
O8	0.0489 (17)	0.092 (2)	0.0561 (19)	−0.0327 (15)	−0.0109 (14)	−0.0076 (15)
P1	0.0494 (6)	0.0432 (5)	0.0414 (6)	−0.0139 (5)	−0.0122 (5)	−0.0112 (4)
P2	0.0369 (5)	0.0489 (6)	0.0498 (6)	−0.0116 (4)	−0.0124 (5)	−0.0204 (5)
S1	0.0443 (6)	0.0740 (7)	0.0502 (7)	−0.0234 (5)	−0.0116 (5)	−0.0123 (5)
S2	0.0501 (6)	0.0683 (7)	0.0440 (6)	−0.0205 (5)	−0.0137 (5)	−0.0118 (5)
S3	0.0464 (6)	0.0618 (6)	0.0438 (6)	−0.0194 (5)	−0.0160 (5)	−0.0070 (5)
S4	0.0426 (6)	0.0695 (7)	0.0497 (6)	−0.0240 (5)	−0.0155 (5)	−0.0085 (5)

### Geometric parameters (Å, °)

**Table:**

C1—C6	1.379 (5)	C33—C34	1.365 (5)
C1—C2	1.396 (5)	C33—H33	0.9300
C1—P1	1.811 (4)	C34—C35	1.366 (6)
C2—C3	1.372 (5)	C34—H34	0.9300
C2—H2	0.9300	C35—C36	1.393 (6)
C3—C4	1.365 (6)	C35—H35	0.9300
C3—H3	0.9300	C36—H36	0.9300
C4—C5	1.364 (6)	C37—C38	1.380 (5)
C4—H4	0.9300	C37—C42	1.385 (5)
C5—C6	1.376 (5)	C37—P2	1.812 (4)
C5—H5	0.9300	C38—C39	1.362 (6)
C6—H6	0.9300	C38—H38	0.9300
C7—C12	1.378 (5)	C39—C40	1.362 (7)
C7—C8	1.380 (5)	C39—H39	0.9300
C7—P1	1.794 (4)	C40—C41	1.350 (7)
C8—C9	1.382 (5)	C40—H40	0.9300
C8—H8	0.9300	C41—C42	1.390 (6)
C9—C10	1.356 (6)	C41—H41	0.9300
C9—H9	0.9300	C42—H42	0.9300
C10—C11	1.369 (6)	C43—C48	1.381 (5)
C10—H10	0.9300	C43—C44	1.395 (5)
C11—C12	1.383 (5)	C43—P2	1.800 (4)
C11—H11	0.9300	C44—C45	1.377 (5)
C12—H12	0.9300	C44—H44	0.9300
C13—C14	1.388 (5)	C45—C46	1.361 (6)
C13—C18	1.389 (5)	C45—H45	0.9300
C13—P1	1.812 (4)	C46—C47	1.372 (6)
C14—C15	1.374 (6)	C46—H46	0.9300
C14—H14	0.9300	C47—C48	1.373 (5)
C15—C16	1.359 (7)	C47—H47	0.9300
C15—H15	0.9300	C48—H48	0.9300
C16—C17	1.371 (7)	C49—N3	1.365 (4)
C16—H16	0.9300	C49—C50	1.393 (5)
C17—C18	1.381 (6)	C49—C54	1.432 (5)
C17—H17	0.9300	C50—C51	1.423 (5)
C18—H18	0.9300	C50—S3	1.761 (4)
C19—N1	1.367 (4)	C51—O5	1.216 (4)
C19—C20	1.396 (5)	C51—O6	1.349 (5)
C19—C24	1.433 (5)	C52—O6	1.492 (7)
C20—C21	1.429 (5)	C52—C53	1.513 (8)
C20—S1	1.760 (4)	C52—H52A	0.9700
C21—O1	1.216 (4)	C52—H52B	0.9700
C21—O2	1.359 (5)	C53—H53A	0.9600
C22—O2	1.456 (5)	C53—H53B	0.9600
C22—C23	1.473 (8)	C53—H53C	0.9600
C22—H22A	0.9700	C52'—O6	1.491 (9)
C22—H22B	0.9700	C52'—C53'	1.523 (10)
C23—H23A	0.9600	C52'—H52C	0.9700
C23—H23B	0.9600	C52'—H52D	0.9700
C23—H23C	0.9600	C53'—H53D	0.9600
C24—C25	1.376 (5)	C53'—H53E	0.9600
C24—C26	1.431 (5)	C53'—H53F	0.9600
C25—S1	1.711 (4)	C54—C55	1.376 (5)
C25—S2	1.716 (4)	C54—C56	1.424 (5)
C26—N2	1.362 (4)	C55—S3	1.709 (4)
C26—C27	1.380 (5)	C55—S4	1.722 (3)
C27—C28	1.443 (5)	C56—N4	1.352 (4)
C27—S2	1.757 (4)	C56—C57	1.392 (5)
N2—H2A	0.8581	C57—C58	1.433 (5)
N2—H2B	0.8612	C57—S4	1.759 (4)
C28—O3	1.213 (4)	N4—H4A	0.8621
C28—O4	1.342 (4)	N4—H4B	0.8573
C29—C30	1.445 (6)	C58—O7	1.225 (4)
C29—O4	1.449 (5)	C58—O8	1.357 (5)
C29—H29A	0.9700	C59—O8	1.455 (5)
C29—H29B	0.9700	C59—C60	1.462 (7)
C30—H30A	0.9600	C59—H59A	0.9700
C30—H30B	0.9600	C59—H59B	0.9700
C30—H30C	0.9600	C60—H60A	0.9600
C31—C32	1.392 (5)	C60—H60B	0.9600
C31—C36	1.393 (5)	C60—H60C	0.9600
C31—P2	1.804 (4)	N1—P1	1.583 (3)
C32—C33	1.373 (5)	N3—P2	1.568 (3)
C32—H32	0.9300		
			
C6—C1—C2	118.4 (4)	C31—C36—H36	120.1
C6—C1—P1	119.7 (3)	C35—C36—H36	120.1
C2—C1—P1	121.7 (3)	C38—C37—C42	117.4 (4)
C3—C2—C1	120.0 (4)	C38—C37—P2	121.1 (3)
C3—C2—H2	120.0	C42—C37—P2	120.9 (3)
C1—C2—H2	120.0	C39—C38—C37	121.5 (4)
C4—C3—C2	120.5 (5)	C39—C38—H38	119.3
C4—C3—H3	119.7	C37—C38—H38	119.3
C2—C3—H3	119.7	C40—C39—C38	120.9 (5)
C5—C4—C3	120.1 (4)	C40—C39—H39	119.6
C5—C4—H4	119.9	C38—C39—H39	119.6
C3—C4—H4	119.9	C41—C40—C39	119.0 (5)
C4—C5—C6	120.1 (5)	C41—C40—H40	120.5
C4—C5—H5	120.0	C39—C40—H40	120.5
C6—C5—H5	120.0	C40—C41—C42	121.2 (5)
C5—C6—C1	120.8 (4)	C40—C41—H41	119.4
C5—C6—H6	119.6	C42—C41—H41	119.4
C1—C6—H6	119.6	C37—C42—C41	120.0 (4)
C12—C7—C8	118.9 (4)	C37—C42—H42	120.0
C12—C7—P1	125.1 (3)	C41—C42—H42	120.0
C8—C7—P1	116.0 (3)	C48—C43—C44	118.5 (4)
C7—C8—C9	119.9 (4)	C48—C43—P2	117.6 (3)
C7—C8—H8	120.1	C44—C43—P2	123.8 (3)
C9—C8—H8	120.1	C45—C44—C43	120.1 (4)
C10—C9—C8	121.0 (4)	C45—C44—H44	120.0
C10—C9—H9	119.5	C43—C44—H44	120.0
C8—C9—H9	119.5	C46—C45—C44	120.3 (4)
C9—C10—C11	119.7 (4)	C46—C45—H45	119.8
C9—C10—H10	120.2	C44—C45—H45	119.8
C11—C10—H10	120.2	C45—C46—C47	120.4 (4)
C10—C11—C12	120.1 (4)	C45—C46—H46	119.8
C10—C11—H11	120.0	C47—C46—H46	119.8
C12—C11—H11	120.0	C46—C47—C48	119.9 (4)
C7—C12—C11	120.5 (4)	C46—C47—H47	120.1
C7—C12—H12	119.8	C48—C47—H47	120.1
C11—C12—H12	119.8	C47—C48—C43	120.8 (4)
C14—C13—C18	118.0 (4)	C47—C48—H48	119.6
C14—C13—P1	119.4 (3)	C43—C48—H48	119.6
C18—C13—P1	122.5 (3)	N3—C49—C50	132.5 (3)
C15—C14—C13	121.6 (4)	N3—C49—C54	117.2 (3)
C15—C14—H14	119.2	C50—C49—C54	110.3 (3)
C13—C14—H14	119.2	C49—C50—C51	128.1 (4)
C16—C15—C14	119.4 (5)	C49—C50—S3	112.4 (3)
C16—C15—H15	120.3	C51—C50—S3	119.6 (3)
C14—C15—H15	120.3	O5—C51—O6	122.4 (4)
C15—C16—C17	120.5 (5)	O5—C51—C50	125.8 (4)
C15—C16—H16	119.7	O6—C51—C50	111.7 (4)
C16—C17—C18	120.5 (5)	O6—C52—C53	103.9 (6)
C16—C17—H17	119.8	O6—C52—H52A	111.0
C18—C17—H17	119.8	C53—C52—H52A	111.0
C17—C18—C13	119.9 (5)	O6—C52—H52B	111.0
C17—C18—H18	120.0	C53—C52—H52B	111.0
C13—C18—H18	120.0	H52A—C52—H52B	109.0
N1—C19—C20	132.6 (3)	O6—C52'—C53'	106.4 (9)
N1—C19—C24	117.7 (3)	O6—C52'—H52C	110.4
C20—C19—C24	109.7 (3)	C53'—C52'—H52C	110.4
C19—C20—C21	128.2 (4)	O6—C52'—H52D	110.4
C19—C20—S1	113.1 (3)	C53'—C52'—H52D	110.4
C21—C20—S1	118.4 (3)	H52C—C52'—H52D	108.6
O1—C21—O2	122.3 (4)	C52'—C53'—H53D	109.5
O1—C21—C20	126.3 (4)	C52'—C53'—H53E	109.5
O2—C21—C20	111.3 (4)	H53D—C53'—H53E	109.5
O2—C22—C23	109.0 (5)	C52'—C53'—H53F	109.5
O2—C22—H22A	109.9	H53D—C53'—H53F	109.5
C23—C22—H22A	109.9	H53E—C53'—H53F	109.5
O2—C22—H22B	109.9	C55—C54—C56	112.9 (3)
C23—C22—H22B	109.9	C55—C54—C49	113.9 (3)
H22A—C22—H22B	108.3	C56—C54—C49	133.3 (3)
C22—C23—H23A	109.5	C54—C55—S3	112.7 (3)
C22—C23—H23B	109.5	C54—C55—S4	113.4 (3)
H23A—C23—H23B	109.5	S3—C55—S4	133.8 (2)
C22—C23—H23C	109.5	N4—C56—C57	125.6 (3)
H23A—C23—H23C	109.5	N4—C56—C54	123.2 (3)
H23B—C23—H23C	109.5	C57—C56—C54	111.2 (3)
C25—C24—C26	112.0 (3)	C56—C57—C58	124.3 (4)
C25—C24—C19	113.9 (3)	C56—C57—S4	112.5 (3)
C26—C24—C19	134.2 (3)	C58—C57—S4	123.1 (3)
C24—C25—S1	113.1 (3)	C56—N4—H4A	115.3
C24—C25—S2	113.9 (3)	C56—N4—H4B	113.5
S1—C25—S2	133.0 (2)	H4A—N4—H4B	130.9
N2—C26—C27	126.4 (3)	O7—C58—O8	123.2 (4)
N2—C26—C24	121.9 (3)	O7—C58—C57	124.1 (4)
C27—C26—C24	111.7 (3)	O8—C58—C57	112.7 (4)
C26—C27—C28	124.4 (3)	O8—C59—C60	109.7 (4)
C26—C27—S2	112.5 (3)	O8—C59—H59A	109.7
C28—C27—S2	123.1 (3)	C60—C59—H59A	109.7
C26—N2—H2A	117.3	O8—C59—H59B	109.7
C26—N2—H2B	115.0	C60—C59—H59B	109.7
H2A—N2—H2B	127.6	H59A—C59—H59B	108.2
O3—C28—O4	123.9 (4)	C59—C60—H60A	109.5
O3—C28—C27	123.9 (4)	C59—C60—H60B	109.5
O4—C28—C27	112.2 (4)	H60A—C60—H60B	109.5
C30—C29—O4	110.2 (4)	C59—C60—H60C	109.5
C30—C29—H29A	109.6	H60A—C60—H60C	109.5
O4—C29—H29A	109.6	H60B—C60—H60C	109.5
C30—C29—H29B	109.6	C19—N1—P1	135.7 (3)
O4—C29—H29B	109.6	C21—O2—C22	116.8 (3)
H29A—C29—H29B	108.1	C28—O4—C29	115.9 (3)
C29—C30—H30A	109.5	C49—N3—P2	139.8 (3)
C29—C30—H30B	109.5	C51—O6—C52'	105.2 (7)
H30A—C30—H30B	109.5	C51—O6—C52	121.7 (8)
C29—C30—H30C	109.5	C52'—O6—C52	30.3 (9)
H30A—C30—H30C	109.5	C58—O8—C59	116.8 (3)
H30B—C30—H30C	109.5	N1—P1—C7	116.42 (16)
C32—C31—C36	118.7 (4)	N1—P1—C1	115.43 (17)
C32—C31—P2	121.5 (3)	C7—P1—C1	111.13 (17)
C36—C31—P2	119.6 (3)	N1—P1—C13	104.62 (17)
C33—C32—C31	120.5 (4)	C7—P1—C13	105.05 (18)
C33—C32—H32	119.7	C1—P1—C13	102.30 (17)
C31—C32—H32	119.7	N3—P2—C43	116.30 (17)
C34—C33—C32	120.3 (4)	N3—P2—C31	116.02 (16)
C34—C33—H33	119.8	C43—P2—C31	111.32 (16)
C32—C33—H33	119.8	N3—P2—C37	102.20 (16)
C33—C34—C35	120.5 (4)	C43—P2—C37	104.06 (16)
C33—C34—H34	119.7	C31—P2—C37	104.99 (18)
C35—C34—H34	119.7	C25—S1—C20	90.27 (18)
C34—C35—C36	120.1 (4)	C25—S2—C27	89.98 (18)
C34—C35—H35	119.9	C55—S3—C50	90.79 (17)
C36—C35—H35	119.9	C55—S4—C57	89.99 (17)
C31—C36—C35	119.7 (4)		
			
C6—C1—C2—C3	−2.6 (6)	C55—C54—C56—N4	179.7 (3)
P1—C1—C2—C3	−177.7 (3)	C49—C54—C56—N4	−1.8 (6)
C1—C2—C3—C4	2.7 (7)	C55—C54—C56—C57	−0.8 (4)
C2—C3—C4—C5	−1.6 (7)	C49—C54—C56—C57	177.7 (3)
C3—C4—C5—C6	0.4 (7)	N4—C56—C57—C58	2.7 (6)
C4—C5—C6—C1	−0.4 (7)	C54—C56—C57—C58	−176.7 (3)
C2—C1—C6—C5	1.5 (6)	N4—C56—C57—S4	−179.5 (3)
P1—C1—C6—C5	176.7 (3)	C54—C56—C57—S4	1.1 (4)
C12—C7—C8—C9	1.4 (6)	C56—C57—C58—O7	1.5 (6)
P1—C7—C8—C9	−177.1 (4)	S4—C57—C58—O7	−176.1 (3)
C7—C8—C9—C10	0.1 (8)	C56—C57—C58—O8	−180.0 (3)
C8—C9—C10—C11	−0.8 (8)	S4—C57—C58—O8	2.4 (5)
C9—C10—C11—C12	0.0 (8)	C20—C19—N1—P1	−19.4 (6)
C8—C7—C12—C11	−2.2 (6)	C24—C19—N1—P1	163.9 (3)
P1—C7—C12—C11	176.2 (3)	O1—C21—O2—C22	−6.6 (6)
C10—C11—C12—C7	1.5 (7)	C20—C21—O2—C22	171.7 (4)
C18—C13—C14—C15	0.1 (6)	C23—C22—O2—C21	−78.1 (6)
P1—C13—C14—C15	−177.5 (3)	O3—C28—O4—C29	−0.1 (6)
C13—C14—C15—C16	−0.3 (7)	C27—C28—O4—C29	−178.5 (3)
C14—C15—C16—C17	0.9 (8)	C30—C29—O4—C28	141.1 (4)
C15—C16—C17—C18	−1.2 (9)	C50—C49—N3—P2	17.1 (7)
C16—C17—C18—C13	1.0 (8)	C54—C49—N3—P2	−162.3 (3)
C14—C13—C18—C17	−0.4 (6)	O5—C51—O6—C52'	19.1 (18)
P1—C13—C18—C17	177.1 (4)	C50—C51—O6—C52'	−161.2 (17)
N1—C19—C20—C21	−4.0 (6)	O5—C51—O6—C52	−8.9 (7)
C24—C19—C20—C21	172.9 (4)	C50—C51—O6—C52	170.9 (5)
N1—C19—C20—S1	−177.7 (3)	C53'—C52'—O6—C51	180 (3)
C24—C19—C20—S1	−0.8 (4)	C53'—C52'—O6—C52	−52.4 (19)
C19—C20—C21—O1	6.6 (7)	C53—C52—O6—C51	107.4 (14)
S1—C20—C21—O1	180.0 (3)	C53—C52—O6—C52'	44 (2)
C19—C20—C21—O2	−171.6 (3)	O7—C58—O8—C59	4.6 (6)
S1—C20—C21—O2	1.8 (4)	C57—C58—O8—C59	−174.0 (3)
N1—C19—C24—C25	178.8 (3)	C60—C59—O8—C58	98.3 (5)
C20—C19—C24—C25	1.4 (4)	C19—N1—P1—C7	−45.8 (4)
N1—C19—C24—C26	−1.6 (5)	C19—N1—P1—C1	87.2 (4)
C20—C19—C24—C26	−179.0 (3)	C19—N1—P1—C13	−161.2 (3)
C26—C24—C25—S1	178.9 (2)	C12—C7—P1—N1	141.8 (3)
C19—C24—C25—S1	−1.4 (4)	C8—C7—P1—N1	−39.8 (4)
C26—C24—C25—S2	0.3 (4)	C12—C7—P1—C1	6.9 (4)
C19—C24—C25—S2	−179.9 (2)	C8—C7—P1—C1	−174.7 (3)
C25—C24—C26—N2	−179.6 (3)	C12—C7—P1—C13	−103.0 (4)
C19—C24—C26—N2	0.8 (6)	C8—C7—P1—C13	75.4 (3)
C25—C24—C26—C27	0.0 (4)	C6—C1—P1—N1	−9.4 (4)
C19—C24—C26—C27	−179.6 (3)	C2—C1—P1—N1	165.7 (3)
N2—C26—C27—C28	1.5 (6)	C6—C1—P1—C7	126.0 (3)
C24—C26—C27—C28	−178.0 (3)	C2—C1—P1—C7	−58.9 (4)
N2—C26—C27—S2	179.2 (3)	C6—C1—P1—C13	−122.3 (3)
C24—C26—C27—S2	−0.3 (4)	C2—C1—P1—C13	52.8 (3)
C26—C27—C28—O3	−1.5 (6)	C14—C13—P1—N1	−43.2 (3)
S2—C27—C28—O3	−178.9 (3)	C18—C13—P1—N1	139.3 (3)
C26—C27—C28—O4	176.9 (3)	C14—C13—P1—C7	−166.4 (3)
S2—C27—C28—O4	−0.6 (5)	C18—C13—P1—C7	16.2 (4)
C36—C31—C32—C33	1.3 (6)	C14—C13—P1—C1	77.5 (3)
P2—C31—C32—C33	178.1 (3)	C18—C13—P1—C1	−100.0 (4)
C31—C32—C33—C34	−1.3 (6)	C49—N3—P2—C43	46.9 (4)
C32—C33—C34—C35	1.1 (7)	C49—N3—P2—C31	−87.0 (4)
C33—C34—C35—C36	−0.9 (7)	C49—N3—P2—C37	159.5 (4)
C32—C31—C36—C35	−1.1 (6)	C48—C43—P2—N3	18.3 (3)
P2—C31—C36—C35	−177.9 (3)	C44—C43—P2—N3	−166.5 (3)
C34—C35—C36—C31	0.9 (7)	C48—C43—P2—C31	154.2 (3)
C42—C37—C38—C39	−0.6 (7)	C44—C43—P2—C31	−30.6 (4)
P2—C37—C38—C39	171.0 (4)	C48—C43—P2—C37	−93.2 (3)
C37—C38—C39—C40	0.1 (8)	C44—C43—P2—C37	82.0 (3)
C38—C39—C40—C41	−0.2 (9)	C32—C31—P2—N3	−158.5 (3)
C39—C40—C41—C42	0.9 (9)	C36—C31—P2—N3	18.2 (4)
C38—C37—C42—C41	1.2 (6)	C32—C31—P2—C43	65.4 (4)
P2—C37—C42—C41	−170.5 (4)	C36—C31—P2—C43	−117.8 (3)
C40—C41—C42—C37	−1.4 (8)	C32—C31—P2—C37	−46.6 (3)
C48—C43—C44—C45	0.5 (6)	C36—C31—P2—C37	130.2 (3)
P2—C43—C44—C45	−174.6 (3)	C38—C37—P2—N3	−91.4 (3)
C43—C44—C45—C46	−0.2 (6)	C42—C37—P2—N3	79.9 (3)
C44—C45—C46—C47	−0.5 (7)	C38—C37—P2—C43	30.0 (4)
C45—C46—C47—C48	0.8 (6)	C42—C37—P2—C43	−158.6 (3)
C46—C47—C48—C43	−0.5 (6)	C38—C37—P2—C31	147.0 (3)
C44—C43—C48—C47	−0.2 (6)	C42—C37—P2—C31	−41.6 (4)
P2—C43—C48—C47	175.3 (3)	C24—C25—S1—C20	0.7 (3)
N3—C49—C50—C51	0.9 (6)	S2—C25—S1—C20	178.9 (3)
C54—C49—C50—C51	−179.7 (3)	C19—C20—S1—C25	0.1 (3)
N3—C49—C50—S3	−179.8 (3)	C21—C20—S1—C25	−174.3 (3)
C54—C49—C50—S3	−0.4 (4)	C24—C25—S2—C27	−0.5 (3)
C49—C50—C51—O5	−1.6 (6)	S1—C25—S2—C27	−178.6 (3)
S3—C50—C51—O5	179.1 (3)	C26—C27—S2—C25	0.5 (3)
C49—C50—C51—O6	178.7 (3)	C28—C27—S2—C25	178.2 (3)
S3—C50—C51—O6	−0.6 (4)	C54—C55—S3—C50	−0.6 (3)
N3—C49—C54—C55	179.4 (3)	S4—C55—S3—C50	178.3 (3)
C50—C49—C54—C55	−0.1 (4)	C49—C50—S3—C55	0.6 (3)
N3—C49—C54—C56	0.9 (5)	C51—C50—S3—C55	180.0 (3)
C50—C49—C54—C56	−178.5 (3)	C54—C55—S4—C57	0.4 (3)
C56—C54—C55—S3	179.3 (2)	S3—C55—S4—C57	−178.6 (3)
C49—C54—C55—S3	0.5 (4)	C56—C57—S4—C55	−0.8 (3)
C56—C54—C55—S4	0.2 (4)	C58—C57—S4—C55	177.0 (3)
C49—C54—C55—S4	−178.6 (2)		

### Hydrogen-bond geometry (Å, °)

**Table:**

*D*—H···*A*	*D*—H	H···*A*	*D*···*A*	*D*—H···*A*
C33—H33···O3	0.93	2.46	3.298 (5)	151
N4—H4B···O7	0.86	2.16	2.817 (4)	134
N4—H4A···N3	0.86	2.29	2.945 (4)	133
N2—H2B···O3	0.86	2.19	2.832 (4)	131
N2—H2A···N1	0.86	2.33	2.965 (4)	131
